# The tethering of chromatin to the nuclear envelope supports nuclear mechanics

**DOI:** 10.1038/ncomms8159

**Published:** 2015-06-15

**Authors:** Sarah M. Schreiner, Peter K. Koo, Yao Zhao, Simon G. J. Mochrie, Megan C. King

**Affiliations:** 1 Department of Cell Biology, Yale School of Medicine, 333 Cedar Street, New Haven, Connecticut 06520-8002, USA; 2 Department of Physics, Yale University, 217 Prospect Street, New Haven, Connecticut 06511, USA; 3 Department of Applied Physics, Yale University, 15 Prospect Street, New Haven, Connecticut 06511, USA

## Abstract

The nuclear lamina is thought to be the primary mechanical defence of the nucleus. However, the lamina is integrated within a network of lipids, proteins and chromatin; the interdependence of this network poses a challenge to defining the individual mechanical contributions of these components. Here, we isolate the role of chromatin in nuclear mechanics by using a system lacking lamins. Using novel imaging analyses, we observe that untethering chromatin from the inner nuclear membrane results in highly deformable nuclei *in vivo*, particularly in response to cytoskeletal forces. Using optical tweezers, we find that isolated nuclei lacking inner nuclear membrane tethers are less stiff than wild-type nuclei and exhibit increased chromatin flow, particularly in frequency ranges that recapitulate the kinetics of cytoskeletal dynamics. We suggest that modulating chromatin flow can define both transient and long-lived changes in nuclear shape that are biologically important and may be altered in disease.

## Abstract (web summary)

The mechanical properties of the metazoan nucleus can be influenced by the nuclear lamina. Here, Schreiner *et al.* show that untethering chromatin from the inner nuclear membrane results in highly deformable, softer nuclei, revealing an important role for chromatin in modulating nuclear mechanics.

## Graphical abstract

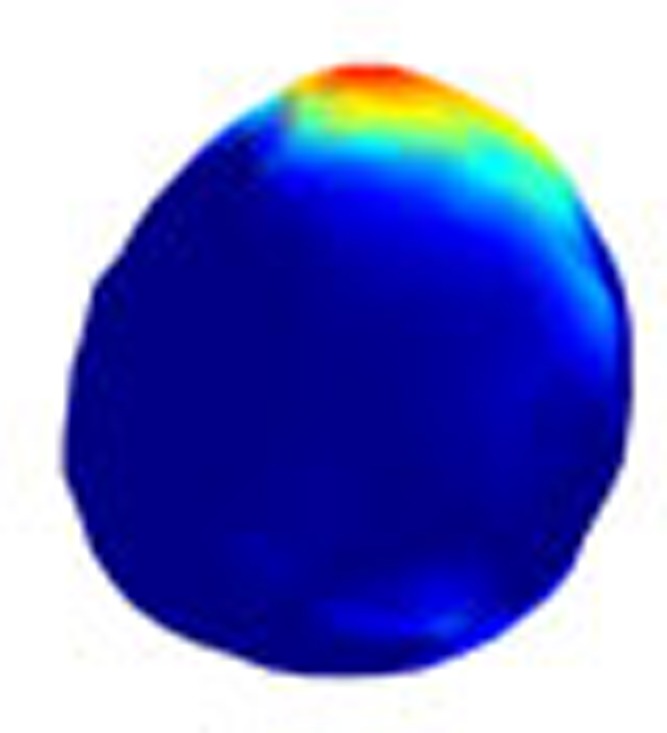

Cells are subjected to mechanical stress, both from extracellular sources (such as forces exerted on the skin or sheer flow across the endothelia of the vasculature) and internal sources (such as forces exerted by the cytoskeleton). Thus, mechanisms to adapt to and dissipate mechanical stress are necessary for cell survival. It is well established that extracellular mechanical forces can be transmitted to the nucleus^1^,^2^. Further, disruption of the mechanical properties of the nucleus during normal cellular processes like migration compromises cell survival^3^,^4^ and can sometimes lead to disease^5^. Defining the factors that underlie the mechanical properties of the nucleus and how these factors can be remodelled is an essential challenge in the field.

The nuclear lamina is considered to be the primary mechanical defense of the mammalian nucleus^6^,^7^. Strong evidence suggests that A-type lamins provide a mechanical buffer to cellular forces in differentiated cells^8^,^9^, particularly those that reside in stiff tissues, whereas the B-type lamins are essential for organogenesis^10^. Mutations in A-type lamins are associated with numerous diseases, including Hutchinson–Gilford progeria syndrome in which nuclei are stiff and fragile^11^,^12^ and Emery–Dreifuss muscular dystrophy in which nuclei are unusually soft^13^. Further, increasing the concentration of lamin A augments the viscoelasticity and rigidity of the nucleus, which has been shown to contribute to mechanical scaling between nuclei and the tissue in which they reside^14^,^15^,^16^.

In addition to the ability of lamins to oligomerize into a polymeric assembly that can directly contribute to the stiffness of the nucleus^6^,^7^,^17^, the lamina is also associated with peripheral heterochromatin^18^,^19^. Indeed, lamins associate with chromatin at the nuclear envelope either through association with integral inner nuclear membrane (INM) proteins such as LAP1 and proteins of the LEM domain family (LAP2, emerin, Man1) or a host of soluble nuclear factors^20^. Mutations in INM proteins, such as emerin and LAP1, underlie a broader set of genetic diseases termed ‘nuclear envelopathies' that in some cases phenocopy lamin mutations^21^. Likely, it is this integrated network of proteins and polymers^18^,^22^ that define the ensemble mechanical properties of the nucleus. This integrated network creates a challenge for dissecting the relative contributions of lamins, INM proteins and the associated chromatin to nuclear mechanics, particularly because of the co-dependence of their localization^23^,^24^.

Unlike lamins, LEM domain proteins were present in the last eukaryotic common ancestor^25^, suggesting that the association of chromatin with the INM is an ancient aspect of nuclear organization. Interestingly, numerous organisms that are largely protected from external forces by a cell wall lack genes homologous to the lamins, suggesting a lamin-independent mechanism capable of buffering internal forces, such as those delivered by the cytoskeleton onto nuclei. Both *Saccharomyces cerevisiae* and *Schizosaccharomyces pombe* express two LEM domain proteins, Heh1 and Heh2 (refs 26, 27, 28). Heh1 and Heh2 are orthologues of the mammalian LEM domain proteins Man1, LEMD2 and emerin^26^,^28^. *S. pombe* has an additional, conserved INM protein, Ima1, which also associates with chromatin^29^ and is homologous to mammalian Net5/Samp1 (refs 29, 30). Importantly, loss of INM proteins leads to apparent defects in nuclear structure in fission yeast^28^,^29^,^31^.

In this study, we investigate how association of chromatin with the nuclear periphery influences the ability of the nucleus to withstand mechanical forces in the model organism, *S. pombe. S. pombe* lacks lamins^25^, allowing us to isolate the contribution of chromatin to nuclear mechanics. In *S. pombe*, the spindle pole body (SPB), the centrosome equivalent in yeast, is tethered to the nuclear envelope (NE) through NE-spanning LINC (Linker of Nucleoskeleton and Cytoskeleton) complexes^32^,^33^,^34^. Microtubules (MTs) emanating from the SPB grow stochastically towards the cell tips^35^; once the growing MT reaches the cell cortex, continued MT polymerization applies a rear-ward force onto the nucleus. This mechanism maintains the nucleus in the middle of the cell to support symmetric cell division^36^. Interestingly, *S. pombe* chromosomes are organized in the ‘Rabl' conformation^37^, with the centromeres clustered at the SPB interface^38^. Thus, centromeric heterochromatin is associated with the region of the NE that is physically linked to dynamic MTs in the cytoplasm.

Here, we use new quantitative approaches to investigate *S. pombe* nuclear mechanics *in vivo* and *in vitro*. Our work demonstrates that the association of chromatin with the INM contributes substantially to nuclear stiffness. Further, increased mobility of chromatin in the absence of INM tethers allows for chromatin flow in response to MT-induced fluctuations, leading to longer-lived defects in nuclear shape. This work suggests that the rate of chromatin flow can be tuned by modulating chromatin tethering to the nuclear periphery.

## Results

### Microtubules drive nuclear envelope fluctuations *in vivo*

To assess the nuclear response to intracellular forces, we first sought to develop an assay to measure NE fluctuations *in vivo*. To visualize the NE, we used strains expressing Cut11-GFP (green fluorescent protein) from its endogenous locus; Cut11 is an integral membrane protein of the nuclear pore complex^39^. Monitoring Cut11-GFP in living cells provided a means to monitor nuclear shape, which responds to thermal forces as well as MT forces *in vivo*^29^,^36^,^39^. We specifically analysed cells in the G2 phase of the cell cycle, which were visually identified on the basis of the criteria that they were single cells (that is, they had completed cytokinesis) and did not have Cut11-GFP associated with the spindle pole body (which occurs at mitotic entry^39^). Three-dimensional (3D) structural information of individual nuclei was encoded in two-dimensional (2D) sequential microscope images collected at different focal depths (10 z-slices each separated by 400 nm) collected every 2.5 s for 5 min.

**Figure 1:**
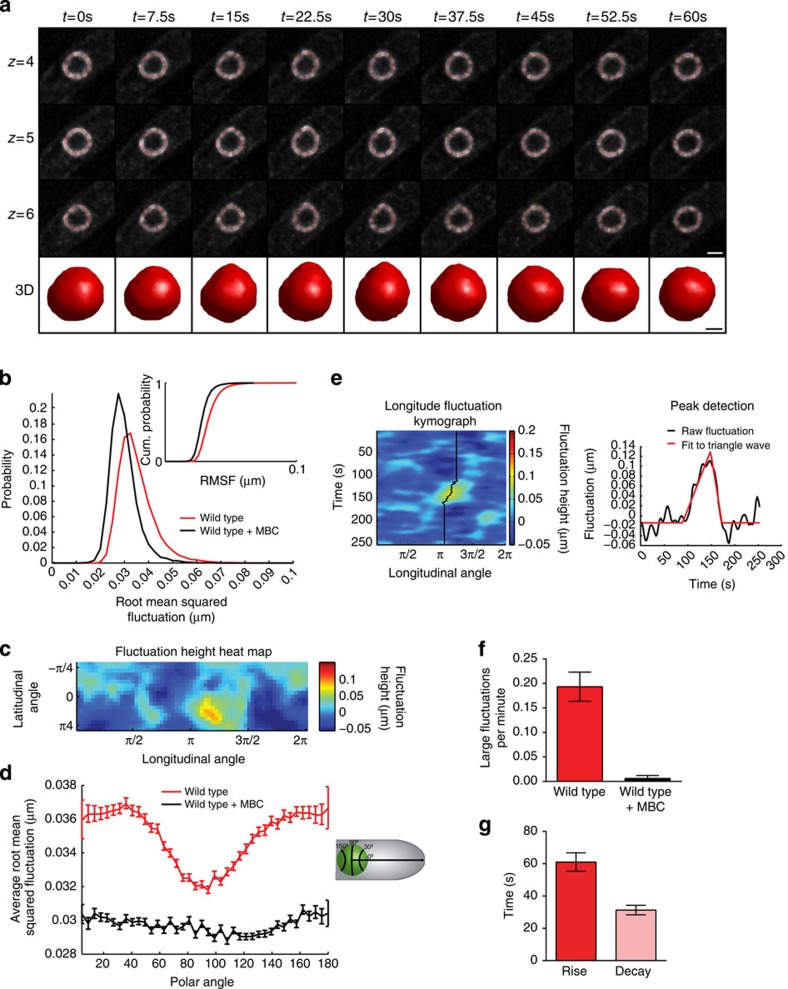
Microtubules drive large nuclear envelope fluctuations *in vivo.* (**a**) Contour surface of a representative wild-type nucleus. The 3D contour of the nucleus was determined at each time point. The 3D contour is projected onto three z-slice images separated by 0.4 μm about the centre of the nucleus over a 60-s time course (top). The fitted 3D contour at each time point is shown below. Scale bars, 1.6 μm (top) and 0.6 μm (bottom). (**b**) Probability distribution of the root mean square fluctuations (RMSF) for wild-type cells (red, *n*=77) and cells treated for 10 min with MBC to depolymerize microtubules (black, *n*=79). Inset: cumulative (cum.) probability distribution of the RMSF. (**c**) The 2D projection map of the 3D NE fluctuations for a representative wild-type nucleus at a given time point. Each pixel represents a fluctuation at each equally spaced angular position along the contour, π/4 latitudinal (polar) angles above and below the centre of the nucleus (*y* axis) and 2π longitudinal (azimuthal) angles around the contour (*x* axis). Fluctuation height is displayed as a heat map, with larger fluctuations in red and smaller fluctuations in dark blue. (**d**) The angular distribution of RMSF averaged over polar angles defined by the cell length in wild-type and wild-type+MBC cells. The coordinate map shows that a zero angle is defined in the direction of the cell length and 90° is in the direction of the cell width (right). (**e**) A kymograph was generated by concatenating the longitudinal pixel line at a fixed latitudinal angle about the centre of a fluctuation at each time point from the 2D fluctuation projection map series (left). The magnitude of the tracked fluctuation at each time point plotted over time (black) is fit with a single asymmetric triangle waveform to determine the rise and decay time (right). (**f**) Quantification of the number of large fluctuations per nuclei per minute in wild-type and wild-type+MBC nuclei. (**g**) Timescales of the rise and decay of microtubule-dependent fluctuations (*n*=31). Plots in **d**, **f** and **g** display mean±s.e.m. Data are from a minimum of three biological replicates.

To quantitatively assess nuclear fluctuations at each time point, we reconstructed a 3D contour of the NE with sub-pixel resolution. We used a novel optimization scheme that seeks to maximize the contour intensity at each image stack while simultaneously minimizing the bending of the contour. A representative reconstruction of a wild-type NE at various time points is shown in Fig. 1a as both a 3D rendering and 2D cross-sections at three z-slices about the centre of the nucleus (see also Supplementary Movie 1). Using spherical coordinates centred on the nucleus at each time point, each NE fluctuation was defined as the difference between the radial coordinate of the contour and its time-averaged radial value; this approach also allows us to correct for changes in nuclear position over time. The frequency and magnitude of fluctuations, sampled at equally spaced spherical angles around the nucleus, were assessed by computing the root mean square fluctuation (RMSF). The RMSF thus provides a quantitative metric for changes in nuclear shape over time.

We compared the RMSF of cells imaged with and without carbendazim (MBC), which depolymerizes MTs. The distribution of RMSF from a population of nuclei over time at each spherical angle clearly shows that untreated nuclei have larger fluctuations on average compared with MBC-treated nuclei (Fig. 1b). Thus, our fluctuation analysis is sufficiently sensitive to capture the impact of active MT dynamics on nuclear shape.

To test whether the actin cytoskeleton also applies forces that can drive fluctuations of the NE, we analysed nuclear fluctuations in cells treated with latrunculin A (Lat A), which depolymerizes actin. Actin depolymerization in *S. pombe* causes a population of cells to arrest in G2 (ref. 40), increasing the size of the cells and, therefore, the size of the nuclei^41^. After applying a size filter to account for this effect (Supplementary Fig. 1a), we found that actin depolymerization in cells caused a minor shift towards larger RMSF values compared with untreated cells (Supplementary Fig. 1b). Thus, MT forces are the primary driver of large NE fluctuations in this system.

To discriminate between fluctuations caused by MT dynamics from thermal fluctuations, we generated a sequence of spatial maps of NE fluctuations (Fig. 1c,e). The landscape of each 3D nuclear contour was obtained by plotting fluctuation size (represented as a heat map) at each latitudinal and longitudinal angle (Fig. 1c). We assessed the angle at which these fluctuations occur using a polar coordinate system based on the long axis of the cell (Fig. 1d). While NE fluctuations in MBC-treated nuclei are small and are uniformly distributed along the NE (Fig. 1d, black line), untreated nuclei have a distinct pattern in which larger NE fluctuations occur at 30 degrees and 150 degrees along the polar axis (Fig. 1d, red line) with a minimum at 90 degrees (orthogonal to the long axis of the cell). Given that MT polymerization is aligned with the long axis of the cell, this result confirms that large NE fluctuations are primarily due to MT-derived forces.

The NE fluctuations maps were next interrogated to spatially track the fluctuation over time, allowing us to monitor the amplitude and duration of a fluctuation event. The one-dimensional angular trajectory of a fluctuation event can be represented with a kymograph (Fig. 1e). The resulting amplitude of each NE fluctuation time series was fit with a single peak asymmetric triangle waveform, allowing us to determine the rise and decay times (Fig. 1e). Comparing untreated versus MBC-treated cells, we established criteria to identify MT-induced fluctuations. These criteria require that a fluctuation maintains a minimum height of 50 nm for a duration longer than 25 s (Supplementary Fig. 1c). This approach was validated by comparing the frequency of fluctuations that meet these criteria in untreated versus MBC-treated cells (Fig. 1f). The duration of large NE fluctuations corresponds very well within the known duration of MT forces on the nucleus (∼90 s) in *S. pombe*^36^ (Fig. 1g). Interestingly, MT-dependent fluctuations decay faster than they form; since MTs in *S. pombe* undergo a rapid catastrophe, the rate at which the fluctuations resolve is likely dependent on the innate physical properties of the nucleus.

### Untethering chromatin alters NE fluctuations *in vivo*

**Figure 2:**
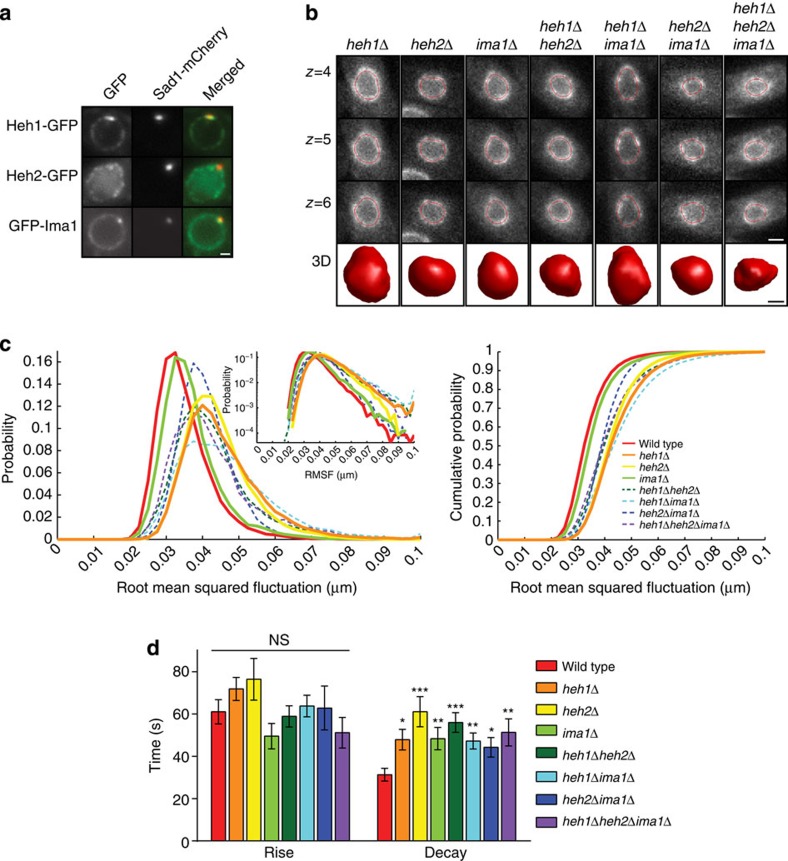
Untethering chromatin from the nuclear envelope affects the nuclear response *in vivo.* (**a**) Representative images of cells expressing Sad1-mCherry and either Heh1-GFP, Heh2-GFP or GFP-Ima1. Scale bar, 0.64 μm. (**b**) Representative 3D contours for each of the INM protein knockout strains as in Fig. 1a. Scale bars, 1.6 μm (top) and 0.6 μm (bottom). (**c**) Comparison of RMSF probability distribution (left) and cumulative probability distribution (right) between wild-type (*n*=77), *heh1Δ* (*n*=61)*, heh2Δ* (*n*=58), *ima1Δ* (*n*=80), *heh1Δheh2Δ (n*=71), *heh1Δima1Δ* (*n*=44), *heh2Δima1Δ* (*n*=53) and *heh1Δheh2Δima1Δ* (*n*=34) nuclei. Wild-type and single mutants are plotted with solid lines and combinations of mutants are shown with dashed lines. Inset—RMSF probability distributions plotted on a log scale to emphasize the tail behaviour of each distribution. (**d**) The average rise time and decay time of microtubule-dependent fluctuations for all strains, measured as in Fig. 1g and plotted as the mean±s.e.m. Wild-type (*n*=31), *heh1Δ* (*n*=52)*, heh2Δ* (*n*=28), *ima1Δ* (*n*=25), *heh1Δheh2Δ (n*=57), *heh1Δima1Δ* (*n*=50), *heh2Δima1Δ* (*n*=18) and *heh1Δheh2Δima1Δ* (*n*=31). NS, not significant, *P<0.05, **P<0.01, ***P<0.001 by Student's *t*-test. Data are from a minimum of three biological replicates.

Our previous work suggests that association of heterochromatin with the INM plays an important role in supporting nuclear mechanics^29^. To quantitatively investigate this model, we focused on three conserved integral INM proteins: Heh1 (also called Lem2), Heh2 (also called Man1) and Ima1, which contribute to chromatin tethering to the nuclear periphery^28^,^42^,^43^,^44^. On the basis of proteomic studies, there are ∼1,000 copies of Heh1, 500 copies of Heh2 and 200 copies of Ima1 per cell^45^. In *S. pombe*, a fraction of Heh1-GFP and GFP-Ima1 co-localize with the *S. pombe* SUN domain protein, Sad1-mCherry, which resides at the centromere–spindle pole body interface of the NE^29^ (Fig. 2a). By contrast, Heh2-GFP is dispersed throughout the NE where it is found in faint foci (Fig. 2a). Importantly, Heh1, Heh2 and Ima1 target independently to the NE and loss of these chromatin tethers does not substantially alter the focal nature of heterochromatin within the nucleus as assessed by visualization of heterochromatin-binding proteins^46^,^47^ (Supplementary Fig. 2b–d). Further, while there are subtle alterations in the total nuclear intensity of heterochromatic foci in *heh1Δ* and *heh2Δ* cells, these differences do not correlate with the observed effects on nuclear mechanics (see below and Supplementary Fig. 2e).

Qualitatively, cells lacking INM proteins have apparent defects in nuclear shape (Fig. 2b and Supplementary Movies 2–8). To quantitatively assess these defects, we compared the RMSF distribution in the absence of individual INM proteins as in Fig. 1. While *ima1*Δ cells display a modest increase in RMSF, both *heh1Δ* and *heh2Δ* cells exhibit a prominent shift towards larger RMSF, with a substantial tail towards very large RMSF values that are rarely observed in wild-type cells (Fig. 2b,c, solid lines). The increased tail of the RMSF distributions seen in *heh1Δ* and *heh2Δ* cells indicates a higher occurrence of very large NE fluctuations.

We next investigated the different combinations of INM protein knockouts. In general, increasing release of chromatin from the INM shifts the distribution to larger RMSF values and further increases the tail of the distribution (Fig. 2b,c, dashed lines). The increase in unusually large NE fluctuations is more clearly revealed when displayed as a semi-log plot (Fig. 2c, inset). Here, it can be seen that all genetic backgrounds that lack Heh1 display a marked increase in very large fluctuations. Importantly, depolymerization of MTs leads to a loss of all large fluctuations for every genotype (Fig. 1b and Supplementary Fig. 3a). Interestingly, the RSMF distributions correlate with the impact of the alleles on growth, with enhancing effects of *heh1* and *ima1* and suppressive effects of *heh2* (Supplementary Fig. 3b). Indeed, *heh1Δheh2Δ* cells show improved growth and smaller RMSF compared with *heh1Δ* cells (Supplementary Fig. 3a,b).

We next investigated whether decoupling chromatin from the nuclear periphery impacted the timescale of NE fluctuations using the same approach as in Fig. 1g. The mean rise time for the deformations was not statistically different between wild-type and cells lacking INM proteins (Fig. 2d), consistent with the expectation that the MT polymerization rate dictates rise time. Interestingly, the decay time of fluctuations was longer in all of the mutant cells compared with wild type. This property appears distinct from the impact on NE fluctuation size. Thus, not only are NE fluctuations generally larger when tethering of chromatin to the nuclear periphery is compromised, but these NE fluctuations also persist longer. Together, these two defects likely underlie the overall changes in nuclear shape that are seen in cells lacking INM proteins (Fig. 2b).

**Figure 3:**
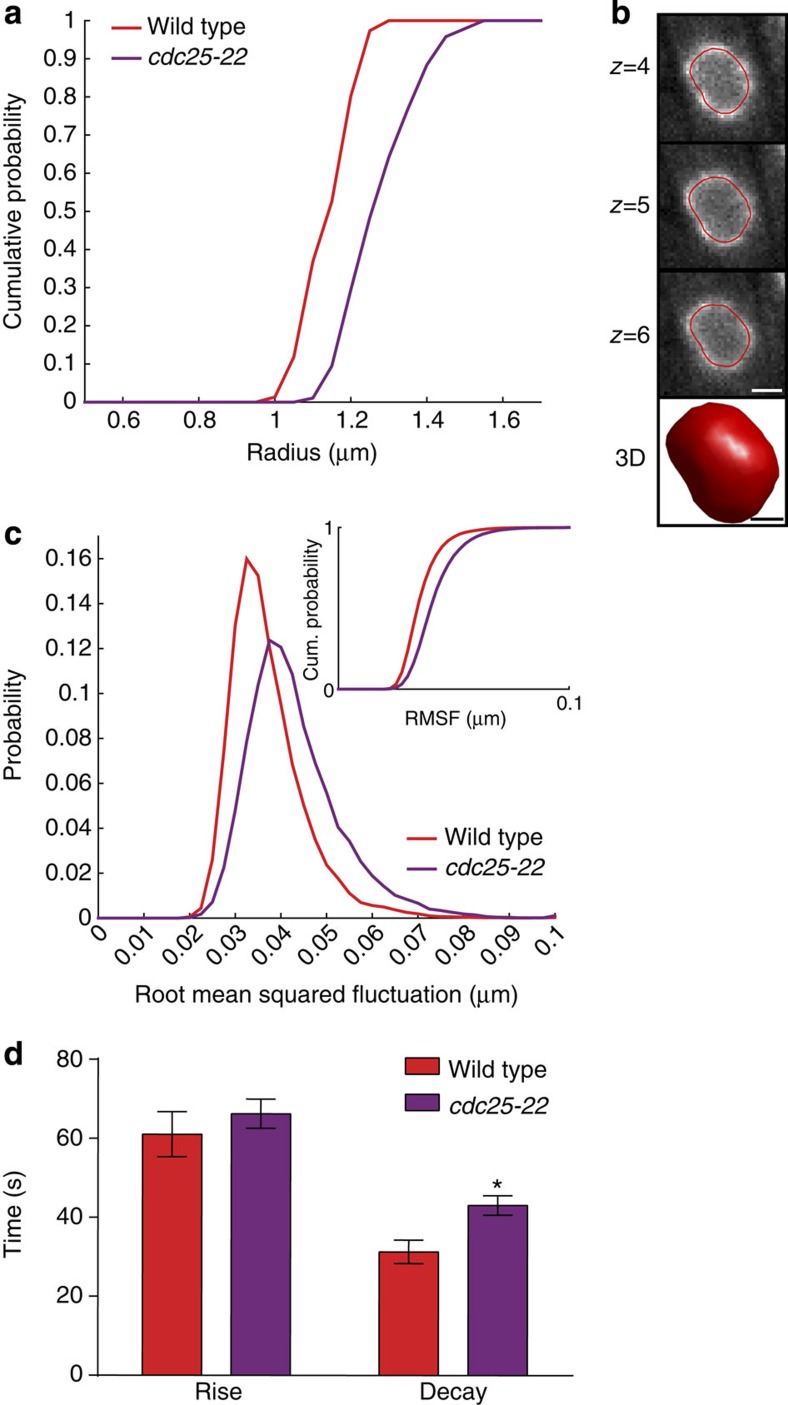
Diluting chromatin tethers at the nuclear envelope moderately affects the nuclear response *in vivo.* (**a**) Cumulative probability plot of nuclear radius for wild-type (red) and *cdc25-22* (purple) cells. (**b**) Representative 3D contour for a *cdc25-22* nucleus as in Fig. 1a. Scale bars, 1.6 μm (top) and 0.6 μm (bottom). (**c**) Comparison of RMSF probability distribution (top) and cumulative (cum.) probability distribution (inset) between wild-type (*n*=77) and *cdc25-22* (*n*=95) nuclei. (**d**) The average rise time and decay time of large fluctuations for wild-type (*n*=31) and *cdc25-22* (*n*=87) nuclei. Plotted as the mean±s.e.m. *P<0.05 by Student's *t*-test. Data are from a minimum of three biological replicates.

We next compared the effect of releasing chromatin from the NE due to loss of chromatin tethers with the effective ‘dilution' of chromatin tethers at the nuclear periphery by increasing nuclear volume. To achieve this, we took advantage of the temperature-sensitive *cdc25-22* strain, which arrests in G2 due to insufficient Cdc2 (Cdk1) activity^48^. During this G2 arrest, the cells continue to grow, leading to a concomitant growth in the nuclear volume^41^ without changes in the amount of chromatin. The *cdc25-22* nuclei have on average an ∼50% increase in their nuclear volume compared with wild-type nuclei (Fig. 3a). Consistent with a model in which chromatin plays a mechanical role at the NE, we find that *cdc25-22* nuclei have both larger RMSF values (although this effect is milder than all but the *ima1Δ* cells) and a slightly longer decay time than wild-type nuclei (Fig. 3b–d and Supplementary Movie 9). This suggests that effective ‘dilution' of chromatin tethers impacts nuclear mechanics, although to a lesser extent than decoupling chromatin from the NE through loss of tethering factors.

Last, we evaluated whether the chromatin state impacts nuclear fluctuation size by treating the cells with trichostatin A (TSA), a histone deacetylase inhibitor. In this condition, we observed abundant tubulation of the NE in both wild-type and INM protein knockout backgrounds (Supplementary Fig. 3c). These tubules disappeared once the cells were treated with MBC, however, MBC-treated nuclei never fully recovered their spherical shape (Supplementary Fig. 3c). While we are cautious to over-interpret these findings given the pleiotropic nature of this perturbation (which leads to extensive changes in gene transcription), these effects suggest that changes in the chromatin state can have a mechanical impact.

### Characterization of wild-type nuclear mechanics *in vitro*

**Figure 4:**
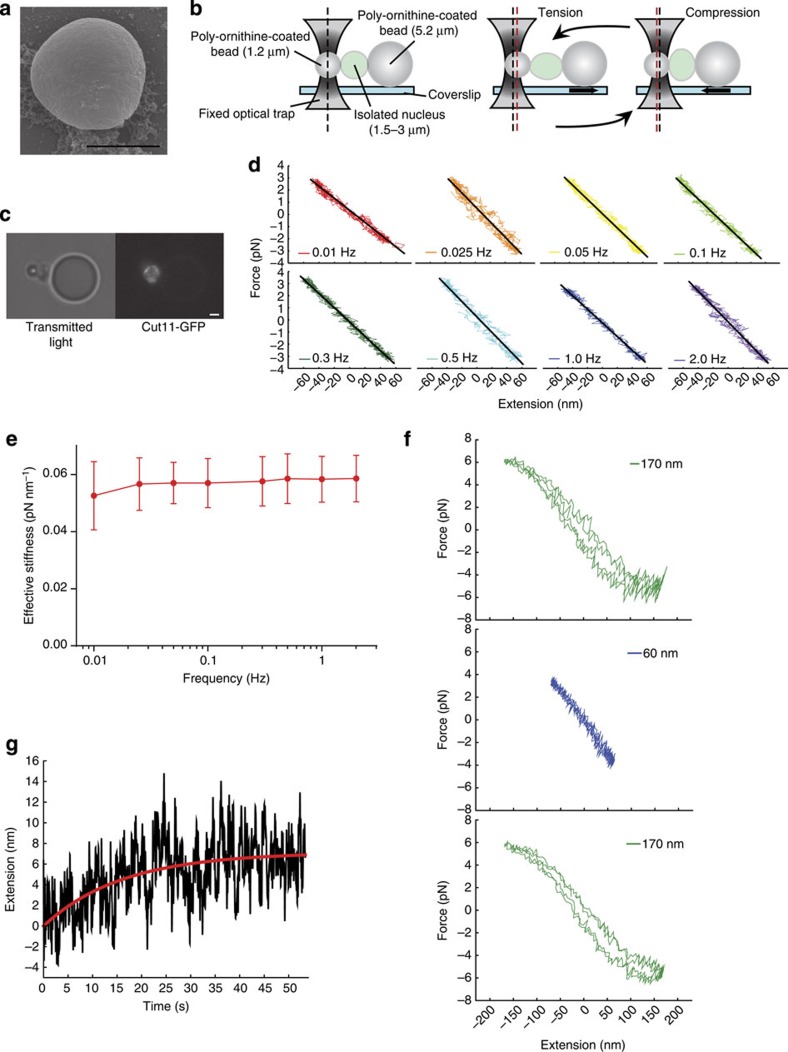
Wild-type nuclei are elastic with a minor viscous component. (**a**) SEM image of an isolated, wild-type nucleus. Scale bar, 2.0 μm. (**b**) Diagram of the *in vitro* optical tweezers assay. An isolated nucleus is adhered to the side of a large (5.2 μm), poly-ornithine-coated bead immobilized on a coverslip. A small (1.2 μm), poly-ornithine-coated bead trapped within a stationary optical trap is attached to the nucleus on the other side. Rounds of tension and compression can be applied to the nucleus by moving the coverslip back and forth sinusoidally. The magnitude of the force applied to the nucleus is measured by the displacement (red vertical dashed line) of the small bead from the centre of the optical trap (black vertical dashed line). (**c**) Representative transmitted light image (left) and wide-field fluorescence image (right) of an isolated, wild-type nucleus expressing Cut11-GFP that is electrostatically attached between a large (5.2 μm) and small (1.2 μm) poly-ornithine-coated bead. Scale bar, 1.1 μm. (**d**) Representative force versus extension relationships and linear fits from one set of 50 nm amplitude oscillation frequencies on a wild-type nucleus. (**e**) Nuclear stiffness of wild-type nuclei from 50 nm (red, *n*=13) amplitude oscillations for a range of frequencies. Two rounds of oscillatory forces were applied at each frequency of a frequency series for each nucleus. Error bars represent s.d. (**f**) Force versus extension relationships for a single nucleus subjected to a set of 170 nm amplitude oscillations (top), followed by 60 nm amplitude oscillations (middle) and another set of 170 nm amplitude oscillations (bottom). (**g**) Representative creep response of a wild-type nucleus acquired via a force clamp, where the extension of the nucleus is constantly adjusted in a feedback loop to maintain a constant 4 pN tensile force. The creep response was fit to Δ*x*(1−exp (−*t*/*τ*)). The best fit parameters for the trace shown are Δ*x*=8.2±0.3 nm and *τ*=16±1.0 s, where the errors represent the square root of the inverse observed Fisher Information. See also Supplementary Fig. 4e.

Although 3D contour fluctuation analysis allows for robust characterization of the nuclear response to MT forces *in vivo*, a full biophysical characterization of nuclear mechanics requires that the nuclear response be measured with calibrated forces across a wide range of timescales *in vitro*. To achieve this, we isolated nuclei from the same strains that were characterized in Fig. 2 by an adaptation of protocols for purification of intact budding yeast nuclei^49^ (see Methods). These nuclei appear intact by scanning electron microscopy (SEM, Fig. 4a) and are impermeable to 70 kDa dextran (Supplementary Fig. 4a). Importantly, these nuclei appear to have the same heterochromatin organization as in intact cells by visualization of GFP-Swi6, the HP1 orthologue (Supplementary Fig. 4b). Nuclei prepared in this manner are sufficiently pure for further experimentation given our ability to select individual nuclei for study (see below).

We developed a novel force spectroscopy assay that allows us to directly measure the stiffness of isolated nuclei under a variety of experimental conditions (Fig. 4b,c). In a flow cell designed with two input ports and one output port (Supplementary Fig. 4c), we nonspecifically adhere large (5.2 μm) poly-ornithine-coated silica beads to the coverslip (Fig. 4b). *S. pombe* nuclei are flowed into the flow cell and an individual nucleus is trapped using the optical tweezers. We select individual nuclei that are between 1.5 and 3 μm, spherical in shape (as assessed by Cut11-GFP fluorescence) and lack any residual tubules of endoplasmic reticulum (visible by transmitted light microscopy, Fig. 4c). Once trapped, each nucleus is associated with the side of one of the large, poly-ornithine-coated silica beads, which elevates the nucleus off of the coverslip and provides a hard wall against which forces can be applied. After the desired nucleus is immobilized, the flow cell design allows the remainder of the nuclear preparation to be washed away by flowing buffer through one of the two input ports. Subsequently, small (1.2 μm) poly-ornithine-coated latex beads are flowed into the flow cell through the second input port, trapped and manoeuvred next to the nucleus. By gently placing the small bead in contact with the nucleus, we are able to induce nonspecific electrostatic interactions between the nucleus and the poly-ornithine-coated surface. Movement of the coverslip (and thereby the large bead and nucleus) while holding the small bead in the optical trap allows us to apply rounds of tension and compression at a set amplitude and frequency, as well as a means to measure the mechanical response of the nucleus (Fig. 4b).

To assess nuclear mechanics over a broad range of timescales, we begin by sinusoidally driving the stage with slow oscillations (0.01–0.5 Hz) that recapitulate the timescale of MT polymerization *in vivo*^36^ and then incrementally increase the frequency of the oscillations on a single nucleus to timescales much faster than MT polymerization rates (1–2 Hz). Importantly, we expended great efforts to improve drift control methods that allow us to obtain reliable data at these biologically relevant timescales, which are often inaccessible by force spectroscopy^50^. We first chose to test oscillation amplitudes that drive 50–60 nm nuclear deformations, which approximately recapitulate the scale of MT-dependent NE fluctuations *in vivo* (Supplementary Fig. 1c). The mechanical response of wild-type nuclei appeared elastic (that is, the force versus extension relationship was linear) with ∼3–4 pN of force at maximal extension at all frequencies (Fig. 4d). Given this linear behaviour, we fit the data to *F*=*k*_nucleus_Δ*x*_nucleus_ where *F* is the force applied, *k*_nucleus_ is the effective stiffness of the nucleus and Δ*x*_nucleus_ is the displacement of the NE from equilibrium^51^. We then compared *k*_nucleus_ for each individual oscillation (Fig. 4e). The nuclei appear to have a very modest frequency-dependent stiffening behaviour, with a slightly softer apparent stiffness between the slowest timescale (0.01 Hz) and all faster timescales (0.025–2 Hz; Fig. 4e). These observations are in line with several mechanical studies of wild-type mammalian nuclei, which also demonstrate time-dependent stiffening behaviour^52^,^53^,^54^. In addition, we tested whether the presence of adenosine triphosphate (ATP) or an ATP regeneration system impacts the mechanical response of the nucleus in this experimental regime, but observed no substantial difference when an individual nucleus was tested sequentially under both conditions (Supplementary Fig. 4d). We chose to carry out the rest of the described experiments in the presence of 2 mM ATP.

The hint of frequency dependence observed for small amplitude oscillations between 0.01 and 0.025 Hz (Fig. 4e) suggests the possibility that wild-type nuclei have a minor viscous component that is not apparent in the individual force versus extension plots (Fig. 4d). To better investigate whether there is such a viscous component to the mechanical response of wild-type nuclei, we applied large oscillatory deformations (∼170 nm), which represent the length scales of rare *in vivo* fluctuations, followed by a series of small oscillatory deformations (∼60 nm), and then another series of large oscillations. At the large oscillations, the force versus extension curves exhibit a reproducible hysteresis, indicating that the nuclei have a minor viscous component (Fig. 4f and Supplementary Fig. 4e). Interestingly, when the compression force reaches ∼4–5 pN, the response of the nucleus begins to plateau. During the intervening small oscillations, we observe the same linear (elastic) response as before (Fig. 4e). As a further test, we also performed force clamp experiments in which we apply a sudden pull on a nucleus with a constant force and monitor the nuclear extension over time (Fig. 4g and Supplementary Fig. 4f). From these experiments, we can access the characteristic timescale of this creep behaviour, which we found is reproducibly in the range of ∼10 s. Interestingly, the characteristic timescale of this viscous component is similar to that described previously for mammalian nuclei^55^. In these studies, it was suggested that the flow of chromatin within the volume of the nucleus underlies a viscous component of nuclear mechanics; such chromatin flow likely explains the mild decrease in apparent stiffness at slow timescales observed here for *S. pombe* nuclei.

### Untethering chromatin alters nuclear mechanics *in vitro*

**Figure 5:**
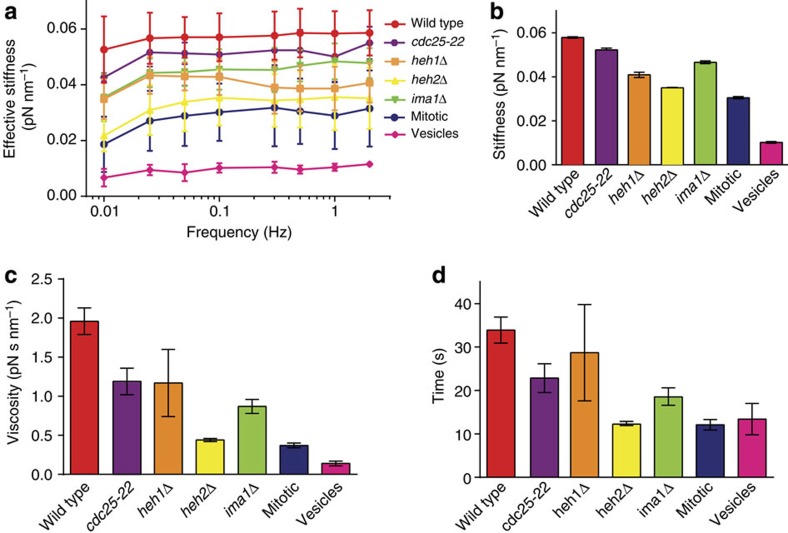
Tethering of chromatin to the nuclear envelope defines nuclear stiffness and viscosity. (**a**) Average nuclear stiffness of wild-type (red, *n*=13), *cdc25-22* (purple, *n*=3), *heh1Δ* (orange, *n*=8), *heh2Δ* (yellow, *n*=7), *ima1Δ* (green, *n*=5) and mitotic nuclei (blue, *n*=4) or vesicles (pink, *n*=3) from 50 nm oscillations over the frequency series. (**b**) Stiffness of the nuclei derived from a viscoelastic fit of the frequency series in **a**. (**c**) Viscosity of the nuclei derived from the viscoelastic fit. (**d**) *τ* derived from the viscoelastic fit. Error bars in all panels represent s.d.

Next, we isolated nuclei lacking individual INM proteins and assessed their mechanical properties using the force spectroscopy assay. Similar to wild-type nuclei, *heh1Δ*, *heh2Δ* and *ima1*Δ nuclei display time-dependent stiffening behaviour at small deformations, which is particularly prominent for *heh2Δ* nuclei (Fig. 5a). This time-dependent effect manifests as an increase in compliance at slow oscillation frequencies (0.01–0.1 Hz). Because this timescale recapitulates the *in vivo* lifetime of NE fluctuations, this suggests that nuclei lacking INM proteins are softest at the frequency range that is most relevant for buffering MT forces *in vivo*. Decreasing the chromatin to nuclear volume ratio also led to a modest decrease in nuclear stiffness at all frequencies compared with wild type (*cdc25-22*, Fig. 5a), consistent with the *in vivo* measurements (Fig. 3).

By fitting the time-dependent stiffening behaviour to a simple viscoelastic model (Supplementary Fig. 4g and Methods), we can clearly see that each INM protein contributes to the observed stiffness of the nucleus, as *heh1Δ*, *ima1*Δ and particularly *heh2Δ* nuclei are all softer than wild type (Fig. 5a,b). Further, wild-type nuclei exhibit higher viscosity than nuclei lacking INM proteins, suggesting that tethering of chromatin to the NE attenuates chromatin flow (Fig. 5a,c). The characteristic timescale *τ*, at which the stiffness plateaus for each type of nucleus, indicates the timescale required for chromatin to respond to external forces. Nuclei lacking INM proteins have shorter *τ* values than wild-type nuclei, further supporting a greater propensity for chromatin to flow in the absence of NE tethers (Fig. 5a,d). By contrast, lowering the chromatin to nuclear volume ratio led to only a slight decrease in stiffness and intermediate value for viscosity and *τ*, despite the fact that *cdc25-22* nuclei having 50% greater volume than wild-type nuclei (Fig. 5c,d). As a control, we applied similar oscillatory forces on vesicles that mimic nuclei in their size, lipid composition and internal viscosity (see Methods); these vesicles are highly deformable relative to all nuclei (Fig. 4a–d).

To further evaluate whether tethering of chromatin to the NE underlies the observed viscous component of nuclear mechanics, we took advantage of the fact that *S. pombe* undergo a closed mitosis in which the NE remains intact but chromatin is globally released from the INM to facilitate chromosome segregation^56^. We allowed cells to accumulate in mitosis by culturing them in the presence of the spindle poison, MBC, followed by isolation of the nuclei. Consistent with our expectation, mitotic nuclei are softer than any of the nuclei lacking individual INM proteins (Fig. 5a,b). Interestingly, they also show clear time-dependent stiffening behaviour (Fig. 5a). By fitting the data to the viscoelastic model, we see that the mitotic nuclei are the most compliant and exhibit the lowest viscosity and shortest characteristic time (*τ*; Fig. 5a–d). This supports a model in which untethering of chromatin from the nuclear periphery decreases both nuclear stiffness and viscosity.

## Discussion

**Figure 6:**
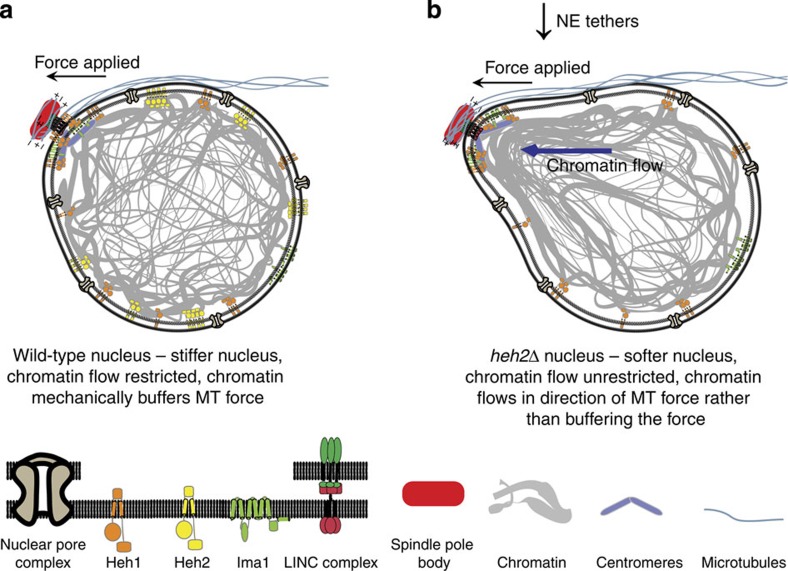
Chromatin tethering to the nuclear periphery impacts stiffness and attenuates chromatin flow. (**a**,**b**) Biological model of the mechanical response of a wild-type nucleus (**a**) and a *heh2Δ* nucleus (**b**) to microtubule forces applied to the spindle pole body. In wild-type nuclei, tethering of chromatin to the nuclear envelope defines nuclear stiffness, preventing large fluctuations and attenuating chromatin flow into the fluctuation. In *heh2Δ* nuclei, the decrease in chromatin tethers leads to a softer nucleus and unrestricted chromatin flow into the fluctuation, leading to larger, longer-lived changes in nuclear shape.

Taken together, these data support a model in which tethering of chromatin to the nuclear periphery supports two critical functions: it imparts stiffness to nuclei and attenuates the flow of chromatin within the nucleus. When stress is delivered onto isolated chromosomes, they respond elastically^57^,^58^. However, within the confines of the nucleus, chromatin can respond to force by either flowing with or bearing the applied stress. Nuclei with a normal extent of chromatin tethers predominantly respond elastically to external forces below 4 pN, suggesting that tethering of chromatin to the NE largely restrains chromatin flow and favours the bearing of stress by chromatin. In this way, the nucleus takes on the mechanical properties of its contents—the chromatin. Untethering of chromatin allows it to flow unabated; this property leads to compromised nuclear stiffness that is particularly weak when nuclei are deformed slowly. While in wild-type nuclei, the characteristic timescale of chromatin flow is sufficiently slow to largely prevent migration of chromatin into MT-dependent deformations (Fig. 6a), the absence of NE chromatin tethers allows chromatin to respond to a deformation by flowing to a new (lower energy) configuration (Fig. 6b). Consequently, residual memory of the deformation persists even after the force is removed, as seen in the slower decay of MT-dependent fluctuations *in vivo* (Fig. 2d). Thus, microtubule bundles impart, on average, deformations smaller than those that impart hysteretic behaviour (Fig. 4f and Supplementary Fig. 4d). This work also provides new insights into how *in vivo* measurements of NE fluctuation size and timescales relate to nuclear mechanics, as nuclear stiffness correlates well with fluctuation size whereas the viscosity correlates well with fluctuation decay time. Further, delineating this relationship may allow insights into nuclear mechanics to be drawn from live-cell imaging rather than requiring complex experimental methods such as force spectroscopy.

The model put forth here suggests that a balance between the biological timescales of nuclear deformations and the rate of chromatin flow defines whether the nucleus retains ‘memory' of previous force. While in wild-type fission yeast such ‘plastic' change appears to be avoided, in other contexts, for example in migrating mammalian cells, physical ‘memory' could facilitate maintaining an altered nuclear shape that supports efficient migration. While it has been observed that A-type lamins impart a viscoelastic property to nuclei in some^14^,^15^, but not all^9^, studies, the association of A-type lamins with chromatin raises the possibility that at least a component of this behaviour is due to the ability of lamins to serve as chromatin tethers, particularly, as we observe viscous behaviour in a system lacking nuclear lamins with decay times on the same order of magnitude as these previous studies^53^,^54^,^59^. Thus, in addition to the clear contribution of the A-type lamin polymer to nuclear stiffness, the ability of the lamina composition to modulate chromatin flow could also play a critical role in establishing the lifetime of changes in nuclear shape. Thus, changes in nuclear shape that are observed in ‘nuclear envelopathies' could arise not just from changes in nuclear stiffness, but also from changes in the kinetics of chromatin flow.

While tethering of chromatin to the NE through INM proteins appears sufficient to support stable nuclear mechanics in response to intracellular forces in *S. pombe*, we expect that these tethering proteins and their associated chromatin are insufficient to provide the stiffness needed for nuclei to remain intact in the face of large forces exerted on tissues (such as the skin) and in those cells that reside within stiff mechanical environments (like bone). However, at different stages of development, the stiffness of mammalian nuclei varies drastically. While stem cell nuclei are soft, increased nuclear stiffness occurs as cells differentiate in conjunction with increasing lamin levels^55^. Thus, tethering of chromatin to the NE may contribute more substantially to the stiffness of stem cells or other cell types that are under low levels of external mechanical stress^7^,^60^. Further studies will be required to investigate how lamins and INM proteins cooperate to define nuclear stiffness and the kinetics of chromatin flow.

Taking into account the measured *in vitro* stiffness of wild-type nuclei (∼0.06 pN nm^−1^) and the average-sized MT-dependent fluctuation from the *in vivo* measurements (∼50–60 nm, Supplementary Fig. 1c), we expect that the microtubule bundle delivers ∼3–4 pN of force on the nucleus, which is within the range possible for MT polymerization from a small number of individual MTs^61^. Interestingly, the yield point at which the elastic response begins to manifest a viscous component, irrespective of the timescales of the applied stress, is about 5 pN (Fig. 4f), which likely exceeds physiological forces; thus, the mechanics of wild-type nuclei support an elastic response *in vivo*. Within this context, the organization of chromatin appears critical. *S. pombe* clusters its centromeres precisely at the region of the nucleus that is subjected to the most powerful MT forces. Accordingly, our findings show that specifically untethering chromatin from the SPB interface (as in *heh1Δima1Δ* cells) leads to the most pronounced mechanical defects *in vivo*. By contrast, loss of Heh2, which is broadly distributed throughout the NE, appears to mildly suppress increased fluctuation size in this context. Such a differential distribution of Heh1 and Heh2 tethers could allow them to act antagonistically, with Heh1 more likely to promote chromatin migration into MT-dependent deformations and Heh2 more likely to restrain this chromatin flow. In our current *in vitro* system, we do not deliver forces specifically onto the centromeric heterochromatin via the SPB and therefore we cannot directly assess this possibility; testing this model will require further assay development.

Interestingly, yeasts are not the only organisms that cluster large blocks of heterochromatin at MT interfaces. Plants, which also do not encode orthologues of the lamin genes, organize centromeres inside the nucleus at sites of MT interaction^62^. In mammalian cells, heterochromatin association with the lamina may, likewise, serve a mechanical role. Such a model suggests new avenues to consider how nuclear mechanics can be modulated during development and in disease.

## Methods

### Cell culture and strain generation

The strains used in this study are listed in Supplementary Table 1. *S. pombe* cells were grown and maintained in standard cell culture conditions^63^. All the strains were grown at 30 °C. Gene replacements were made by replacement of the open reading frames with the kanMX6 (ref. 64), hphMX6 or natMX6 (ref. 65) cassettes. C-terminal GFP tagging was performed with pFa6a-GFP-kanMX6 (ref. 64). The pFa6a-mCherry-kanMX6 cassette was used as a template for C-terminal mCherry tagging^66^. N-terminal GFP tagging was performed as established using pFa6a-kanMX6-nmt41-GFP^64^. All strains generated by cassette integration were confirmed by PCR. Strains made through genetic crosses were confirmed by the segregation of markers and/or by the presence of the appropriate fluorescently tagged protein.

### Growth curves

For growth assays, 5 ml cultures were grown in YE5S at 30 °C overnight to saturation. Cultures were diluted to an OD_600_ ∼0.05 in 50 ml of YE5S and recovered at 30 °C for 2–3 h. OD_600_ measurements were taken every hour for 8 h. Doubling time was found using *T*=log(2*t*_2_*−t*_1_)/log(*y*/*x*), where *T*=generation time, *y*=cells per ml at *t*_2_ and *x*=cells per ml at *t*_1_. Cells per ml were calculated using the following equation: *x*=(2 × 10^6^)/(0.1 × OD_600_).

### Microscopy

*S. pombe* strains were grown in YE5S plus 250 mg l^−1^ adenine to log phase (OD_600_ 0.5–0.8). Cells were mounted on agarose pads (1.4% agarose in Edinburgh minimal media) and sealed with VALAP (1:1:1, vaseline:lanolin:paraffin). Cells treated with MBC were incubated on agar pads with 50 μg ml^−1^ MBC for 10 min before imaging. Cells treated with TSA were grown in YE5S media plus 30 μg ml^−1^ TSA overnight at 30 °C and placed on agar pads with 30 μg ml^−1^ TSA for imaging. Cells treated with Lat A were grown to log phase and treated with 100 μM Lat A 30 min before imaging and placed on agar pads with 100 μM Lat A for imaging. Live-cell images were acquired on a Deltavision Widefield Deconvolution Microscope (Applied Precision/GE Healthcare) with a CoolSnap HQ2 CCD camera (Photometrics) or an Evolve 512 EMCCD camera (Photometrics). For nuclear contour imaging, 10 z-slices with 400 nm spacing were taken every 2.5 s for 10 min with the EMCCD camera. For INM protein localization imaging, 10 z-slices with 200 nm spacing were taken with the CCD camera. A representative z-slice from the image stack was chosen for figures. For intensity measurements of Chp1-GFP, we used a 3D Gaussian function to fit each focus. The values of the number of foci and integrated intensity from each cell so-obtained are presented in Supplementary Fig. 2e.

### Contour fitting

We developed an optimization procedure that robustly reconstructs the shape of the nucleus in three spatial dimensions with sub-pixel resolution based on a stack of 10 z-plane 2D images of the NE separated by 400 nm. Our algorithm seeks to find the contour that maximizes the sum of two terms, namely a term corresponding to the total intensity of the NE across the z-stack, *I*(*r*,*θ*,*ϕ*), and a term corresponding to the negative of the total curvature of the NE, given according to:


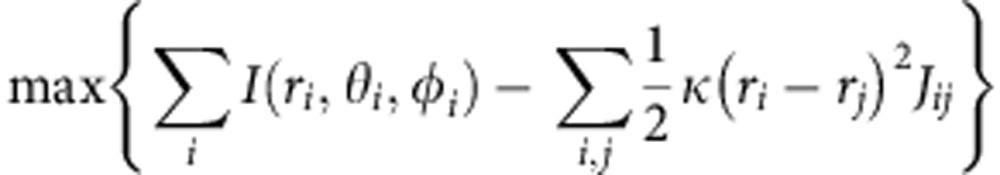


where the sum is over equally spaced angular vertices, *κ* is the computational bending constant, and *J*_*ij*_ is equal to 1 for nearest-neighbour vertices and 0 otherwise. The bending constant of the NE was set automatically via an algorithm that uses the measured intensities to impose a smoothness constraint on the NE, thus eliminating spurious fluctuations. Hence, bright pixels provide a better definition of the contour while noise, caused by a low signal-to-noise ratio, is properly interpolated in dim membrane regions. Although our procedure provides a closed surface of the nuclear contour, the reconstructed cap regions of the nucleus suffer from larger noise for several reasons: the distance between each z-stack interval is larger than the image pixel size, the point spread function is wider in the *z* axis, the aberration of the microscope becomes progressively worse for z-slices away from the focus and the drift of images between z-slices. To minimize these noise contributions, we chose to proceed with our fluctuation analysis by only including a band around the centre of the nucleus defined by 45 degrees above and below the centre stack.

To decouple the motions of the nucleus due to dynamic fluctuations from overall motions of the nucleus, we apply a low-pass Butterworth filter on the centroid position of the nucleus and set these positions as the origin of the spherical coordinate system at each time point. The radius at equally spaced spatial angles from the centre of the nucleus define the positions of the NE at time *t* according to *r*(*θ*,*φ*,*t*). Code available upon request.

### Contour analysis

To quantify the changes in contour shape, we use the RMSF calculated according to:


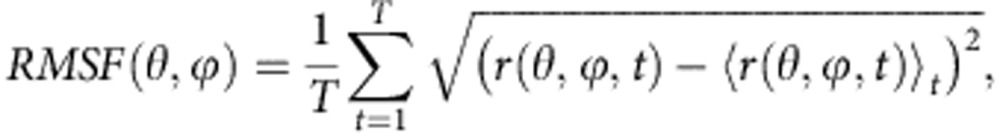


where *T* is the total number of frames, *r*(*θ*,*ϕ*,*t*) is the radial distance from the centre of the contour at vertex angle (*θ*,*ϕ*) at frame *t* and 〈*r*(*θ*,*ϕ*,*t*)〉_*t*_ is the time-averaged radial distance at vertex angle (*θ*,*ϕ*).

### Fluctuation time analysis

We identified all the large fluctuation events using a 2D single particle tracking technique. We first projected the fluctuations onto a 2D spatial map, where the *x* axis corresponds to a different longitudinal angle (0, 2π) and the *y* axis corresponds to a different longitudinal angle and the *y* axis corresponds to a different latitudinal angle (−π/4, π/4). In this representation, a fluctuation can be approximated as a 2D Gaussian profile. We identified large fluctuations that have fitted amplitudes >50 nm in each fluctuation map over time. We next applied a single particle tracking algorithm to connect the locations of the large fluctuations across sequential frames^67^. We only analyse trajectories that last longer than 10 frames (25 s) to filter out short-lived fluctuations, which we ascribe to spontaneous, thermal fluctuations of the NE (validated by the analysis in Supplementary Fig. 1c). The magnitude of each fluctuation trajectory is then fit to a single asymmetric triangle waveform to determine the rise time, decay time and peak height.

### Nuclear isolation

Nuclei were isolated by modification of an established protocol described for *S. cerevisiae*^49^. Strains were grown to log phase at 30 °C in YE5S and diluted to an OD_600_ of 0.01 in 1 liter YE5S. After growth overnight, cells were collected at an OD_600_ between 0.8 and 1.0. To prepare for spheroplasting, cells were incubated in 100 mM Tris pH 9.4 and 10 mM DTT for 10 min at 33 °C. Cells were spheroplasted in 0.4 mg ml^−1^ zymolyase (MP Biomedicals), 0.6 mg ml^−1^ lysing enzymes (Sigma), 350 μl of beta-glucuronidase (MP Biomedicals) and 5 mM DTT in 1.1 M sorbitol. Spheroplasting was allowed to proceed at 33 °C for 2–3 h. Spheroplasts were isolated from intact cells by collection through a cushion of 7.5% Ficoll in 1.1 M sorbitol spun in a SW28 rotor (Beckman) at 10,000*g* for 15 min at 4 °C. Cells were lysed by gentle homogenization in 0.25% Surfact-Amps X-100 (Thermo), 5 mM DTT, 200 μl protease inhibitor cocktail (Sigma), 0.2 mg ml^−1^ PMSF, 4 μg ml^−1^ Pepstatin A (American Bio) in 8% polyvinylpyrrolidone (Sigma). Three density-step sucrose gradients were prepared at the following concentrations: 1.875, 2.30 and 2.50 M. To purify nuclei, lysates were applied to the sucrose gradient and spun in a SW28 rotor (Beckman) at 14,118*g* for 90 min at 4 °C. Isolated nuclei sedimented at the interface between the 1.875 and 2.3 M sucrose layers. 100 μl aliquots were flash frozen in liquid N_2_ and stored at −80 °C. For use, isolated nuclei aliquots were dialysed in 20,000 MWCO Slide-A-Lyzer MINI Dialysis Units (Thermo) overnight into 500 ml dialysis buffer (80 mM PIPES, 5% DMSO, 2 mM MgCl_2_, 1 mM EGTA, 500 mM sucrose). Nuclear integrity was confirmed by incubating the nuclei in 10 μg ml^−1^ FITC 70 kD Dextran (Sigma) and testing for nuclear exclusion.

### SEM sample preparation and imaging

SEM samples were prepared on the basis of previously published methods^68^. Silicon chips (5 × 5 mm^2^) were prepared by incubating in 1 mg ml^−1^ poly-L-lysine at room temperature for 30 min. Silicon chips were rinsed with Fixative-1 (4% paraformaldehyde, 20 mM potassium phosphate pH 6.5, 0.5 mM MgCl_2_, 0.2 M sucrose) and placed into an inverted microcentrifuge tube lid separated from the top of the tube. The lid with silicon chip was then placed at the base of a microcentrifuge tube with its bottom cut off. The chip was then overlayed with 20 μl of Fixative-1. Dialyzed, isolated nuclei (8 μl) were placed on top of the fixative in the microcentrifuge chamber. To bind the nuclei to the silicon chip, the microcentrifuge chamber was spun at 1,000*g* for 3 min at 4 °C. The silicon chips were then removed from the chamber and rinsed with Fixative-1 without sucrose and incubated in Fixative-2 (2% glutaraldehyde, 0.2% tannic acid in 20 mM potassium phosphate, 0.5 MgCl_2_, pH 7.4) for 10 min at room temperature. Silicon chips were rinsed with dH_2_O and fixed in 1% osmium tetroxide for 10 min. Samples were dehydrated by incubating for 2 min in each 30%, 50%, 70%, 95% (twice) and 100% (three times) ethanol in series. After final dehydration in 100% ethanol, the samples were dried using a Polaron critical point dryer with liquid carbon dioxide as transitional fluid. The silicon chips were glued to aluminium stubs and sputter coated with 15 nm gold (Electron Microscopy Science). The samples were viewed and digital images acquired in an FEI ESEM between 10 kV at a working distance of 10 mm.

### Vesicle preparation

Vesicles were prepared on the basis of methods previously described^69^ and all lipids were purchased from Avanti. A 20:10:70 ratio of lipids DOPE (1,2-dioleoyl-*sn*-glycero-3-phosphoethanolamine), DOPS (1,2-dioleoyl-*sn-*glycero-3-phospho-L-serine) and DOPC (1,2-dioleoyl-*sn*-glycero-3-phosphocholine), respectively were dissolved in chloroform and dried to a film under nitrogen gas. The mixed lipids were resuspended in vesicle buffer (80 mM PIPES pH 6.8, 1 mM MgCl_2,_ 1 mM EGTA, 170 mM sucrose, 1 mg ml^−1^ 70 kD FITC dextran) to 10–30 mM and subjected to seven freeze–thaw cycles. Resuspended lipids were extruded through two polycarbonate membranes (Whatman) with 3.0 μm pore size using the LipSoFast-Basic extruder (Avestin) at room temperature and stored at −80 °C. For use, vesicles were diluted in nuclei dialysis buffer minus sucrose and spun at 10,000*g* in a TLA 100.3 (Beckman) rotor for 30 min at 4 °C. Vesicles were resuspended in 500 μl dialysis buffer with sucrose for experiments.

### Optical tweezers sample preparation

Flow cells were constructed by cutting double-sided sticky tape to create a flow channel between a microscope coverslip and a microscope slide that contains holes to allow for two inlet ports and one outlet port. Dry, 5.20 μm silica-NH_2_ microspheres (Bangs Laboratories) were resuspended in 100% EtOH. A concentrated slurry of microspheres was added to 170 μl of 0.01% poly-L-ornithine (Sigma), loaded into the flow cell and incubated for a minimum of 2 h. Flow cells were then rinsed with dialysis buffer. Aliphatic amine latex beads (2% w/v 1.2 μm, Invitrogen) were pre-incubated for a minimum of 2 h in poly-l-ornithine, then rinsed with dialysis buffer. Nuclei were loaded onto flow cell immediately before experiments. Latex beads were diluted 1:300 into dialysis buffer+2 mM ATP. The dialysis buffer+2 mM ATP and the latex bead mixture were loaded into separate syringes and connected to the two inlet ports.

### Optical tweezers setup

We use a single-beam gradient optical trap for both trapping and back focal plane interferometry position detection. The trapping beam, produced by a 3 W 1,064 nm ND:YAG laser (Laser Quantum, Ventus IR), passes through an acousto-optic deflector (IntraAction) to control the position and intensity of the laser beam and also acts as an optical isolator. The beam is then expanded by a factor of 3 to slightly overfill the back aperture of the objective (Nikon, CFI × 100, 1.2 NA). The focused laser is used to trap a bead inside of a flow cell, which rests on a custom-built sample stage holder mounted on a Thorlabs nanomax positioner, consisting of coarse stage motors with micron scale accuracy and a piezo stage with nanometre resolution. The forward scattered light is collected with a condenser and is directed onto an infrared-enhanced quadrant photodiode (First Sensor) located in a conjugate plane to the back focal plane of the condenser. To visualize the nucleus via fluorescence, an epi-fluorescence microscope is coupled into the optical tweezers setup. The excitation source, produced by a 470 nm blue light emitting diode (Luxeon Star), is reflected with a long pass dichroic mirror (Semrock) to the back of the objective. The backscattered fluorescent light is collected with the same objective and is transmitted through a dichroic mirror and two bandpass filters (Semrock) placed in series to attenuate non-fluorescence light, including backscattered light from the trapping laser. The fluorescence signal then travels through a tube lens onto an Orca R2 CCD camera (Hamamatsu). All instrument controls and data acquisition is accomplished with custom-made virtual instruments programmed in Labview (National Instruments).

### Optical tweezers oscillation assay

Isolated nuclei were flowed into the flow cell and an individual nucleus was trapped using the optical tweezers and manipulated onto the side of a surface-immobilized 5.20 μm silica-NH_2_ microsphere. The nuclei associate with the microspheres via electrostatic interactions. Excess nuclei were rinsed away by flowing in dialysis buffer+2 mM ATP. Poly-ornithine-coated latex beads (1.20 μm) were loaded into the flow cell and a single bead was trapped with the optical tweezers. The trapped bead was brought close to the nucleus opposite of the large bead-nucleus interface (see diagram in Fig. 4). As the trapped bead was brought into contact with the nucleus, we monitored its displacement within the optical trap, which allowed us to determine the equilibrium position of the nucleus. Upon contact, electrostatic interactions adhere the latex bead with the nucleus. Excess beads were rinsed away with dialysis buffer +2 mM ATP. The stage was driven sinusoidally with an amplitude of ∼60–70 nm or ∼150–170 nm to apply rounds of compression and tension forces on the nucleus at different frequencies, as indicated.

### Drift control

To enable high-precision measurements for extended durations, we have implemented a drift correction procedure^50^. Specifically, two template images are acquired, both consisting of a window about an isolated surface-attached bead. One image is in focus. The other image is out of focus, displaced from the in-focus condition by a known distance along the beam direction (*z*). To correct for in-plane (*xy* plane) drift, the *x* and *y* axes of the piezo stage are adjusted to maintain the maximum cross-correlation between the in-focus template image and the corresponding measured image at subsequent times. Thus, we are able to stabilize the position within the *xy* plane to within 1 nm. To correct for drift along *z*, we exploit the observation that the maximum cross-correlation between the out-of-focus template image and the measured image varies linearly with their *z* displacement in the relevant range. Therefore, we correct *z*-drift by holding fixed the value of the maximum cross-correlation between the out-of-focus template image and the measured image via a PID controller implemented in LabView. In this way, we stabilize the *z*-position to within 2.5 nm. Key to the success of our approach is the use of partially coherent illumination from an LED such that fringes are a prominent feature of the bead images and depend sensitively on the *z*-position.

### Optical tweezers analysis

The effective stiffness of the nucleus, *k*_nucleus_, is determined by applying a linear fit to *F*=*k*_nucleus_Δ*x*_nucleus_, where Δ*x*_nucleus_ is the extension of the nucleus from equilibrium. By exploiting the fact that the force on the nucleus is equal and opposite to the force on the trapped bead, the force on the nucleus is determined by *F=k*_trap_Δ*x*_trap_, where Δ*x*_trap_ is the displacement of the bead from the centre of the trap, and *k*_trap_ is the optical trap stiffness. The stiffness of the optical trap is found by fitting the power spectral density of a trapped 1.2 μm latex bead to a Lorentzian function to determine the corner frequency^70^. The stiffness is then determined as the ratio of the corner frequency to the bead's friction coefficient, corrected using Faxen's Law to account for the proximity of the glass coverslip. Δ*x*_trap_ is determined by a quadrant photodiode, which is calibrated by scanning the trapping laser over a surface-immobilized bead^70^. The extension of the nucleus from equilibrium is determined as the difference between the displacement of the piezo stage from equilibrium and the displacement of the bead from the centre of the trap, according to Δ*x*_nucleus_=Δ*x*_piezostage_−Δ*x*_trap_.

### Viscoelastic model

We fit the frequency dependence of the effective stiffness of the nucleus with a Maxwell viscoelastic model in which a spring and dashpot in series captures the elastic component, *K*, and viscous component, *η*, respectively. The corresponding fitting function for the effective stiffness is given according to:


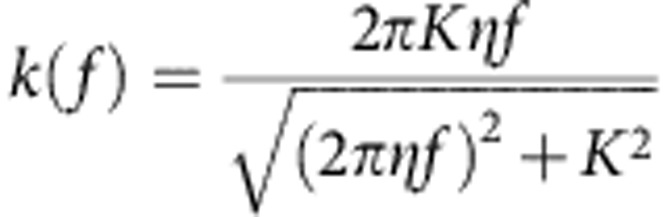


and the characteristic time is given according to


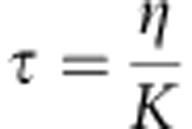


## Additional information

**How to cite this article:** Schreiner, S.M. *et al.* The tethering of chromatin to the nuclear envelope supports nuclear mechanics. *Nat. Commun.* 6:7159 doi: 10.1038/ncomms8159 (2015).

## Supplementary Material

Supplementary Information Supplementary Figures 1-4 and Supplementary Tables 1-2

Supplementary Movie 1 2D cross-sections at three z-slices about the center of the nucleus (right) and the corresponding 3D rendering (left) of the wild type nucleus from Figure 1a. Cells were imaged every 2.5s for 5 minutes. Movies shown at 3 frames/second. Scale bar equals 1 μm in 2D cross-sections. Nuclear deformations are highlighted using the heat map shown in the movie.

Supplementary Movie 2 2D cross-sections at three z-slices about the center of the nucleus (right) and the corresponding 3D rendering (left) of the heh1Δ nucleus from Figure 2b. Cells were imaged every 2.5 seconds for 5 minutes. Movies shown at 3 frames per second. Scale bar equals 1 μm in 2D cross-sections. Nuclear deformations are highlighted using the heat map shown in the movie.

Supplementary Movie 3 2D cross-sections at three z-slices about the center of the nucleus (right) and the corresponding 3D rendering (left) of the heh2Δ nucleus from Figure 2b. Cells were imaged every 2.5 seconds for 5 minutes. Movies shown at 3 frames per second. Scale bar equals 1 μm in 2D crosssections. Nuclear deformations are highlighted using the heat map shown in the movie.

Supplementary Movie 4 2D cross-sections at three z-slices about the center of the nucleus (right) and the corresponding 3D rendering (left) of the ima1Δ nucleus from Figure 2b. Cells were imaged every 2.5 seconds for 5 minutes. Movies shown at 3 frames per second. Scale bar equals 1 μm in 2D crosssections. Nuclear deformations are highlighted using the heat map shown in the movie.

Supplementary Movie 5 2D cross-sections at three z-slices about the center of the nucleus (right) and the corresponding 3D rendering (left) of the heh1Δheh2Δ nucleus from Figure 2b. Cells were imaged every 2.5 seconds for 5 minutes. Movies shown at 3 frames per second. Scale bar equals 1 μm in 2D cross-sections. Nuclear deformations are highlighted using the heat map shown in the movie.

Supplementary Movie 6 2D cross-sections at three z-slices about the center of the nucleus (right) and the corresponding 3D rendering (left) of the heh1Δima1Δ nucleus from Figure 2b. Cells were imaged every 2.5 seconds for 5 minutes. Movies shown at 3 frames per second. Scale bar equals 1 μm in 2D cross-sections. Nuclear deformations are highlighted using the heat map shown in the movie.

Supplementary Movie 7 2D cross-sections at three z-slices about the center of the nucleus (right) and the corresponding 3D rendering (left) of the heh2Δima1Δ nucleus from Figure 2b. Cells were imaged every 2.5 seconds for 5 minutes. Movies shown at 3 frames per second. Scale bar equals 1 μm in 2D cross-sections. Nuclear deformations are highlighted using the heat map shown in the movie.

Supplementary Movie 8 2D cross-sections at three z-slices about the center of the nucleus (right) and the corresponding 3D rendering (left) of the heh1Δheh2Δima1Δ nucleus from Figure 2b. Cells were imaged every 2.5 second for 5 minutes. Movies shown at 3 frames per second. Scale bar equals 1 μm in 2D cross-sections. Nuclear deformations are highlighted using the heat map shown in the movie.

Supplementary Movie 9 2D cross-sections at three z-slices about the center of the nucleus (right) and the corresponding 3D rendering (left) of the cdc25-22 nucleus from Figure 3b. Cells were imaged every 2.5 second for 5 minutes. Movies shown at 3 frames per second. Scale bar equals 1 μm in 2D crosssections. Nuclear deformations are highlighted using the heat map shown in the movie.
