# Supplementary material for: The tethering of chromatin to the nuclear envelope supports nuclear mechanics
Source: Nat Commun. 2015 Jun 15;6:7159. doi: 10.1038/ncomms8159 (PMC4490570; doi:10.1038/ncomms8159)
Supplement: Supplementary Information — Supplementary Figures 1-4 and Supplementary Tables 1-2 [file ncomms8159-s1.pdf]

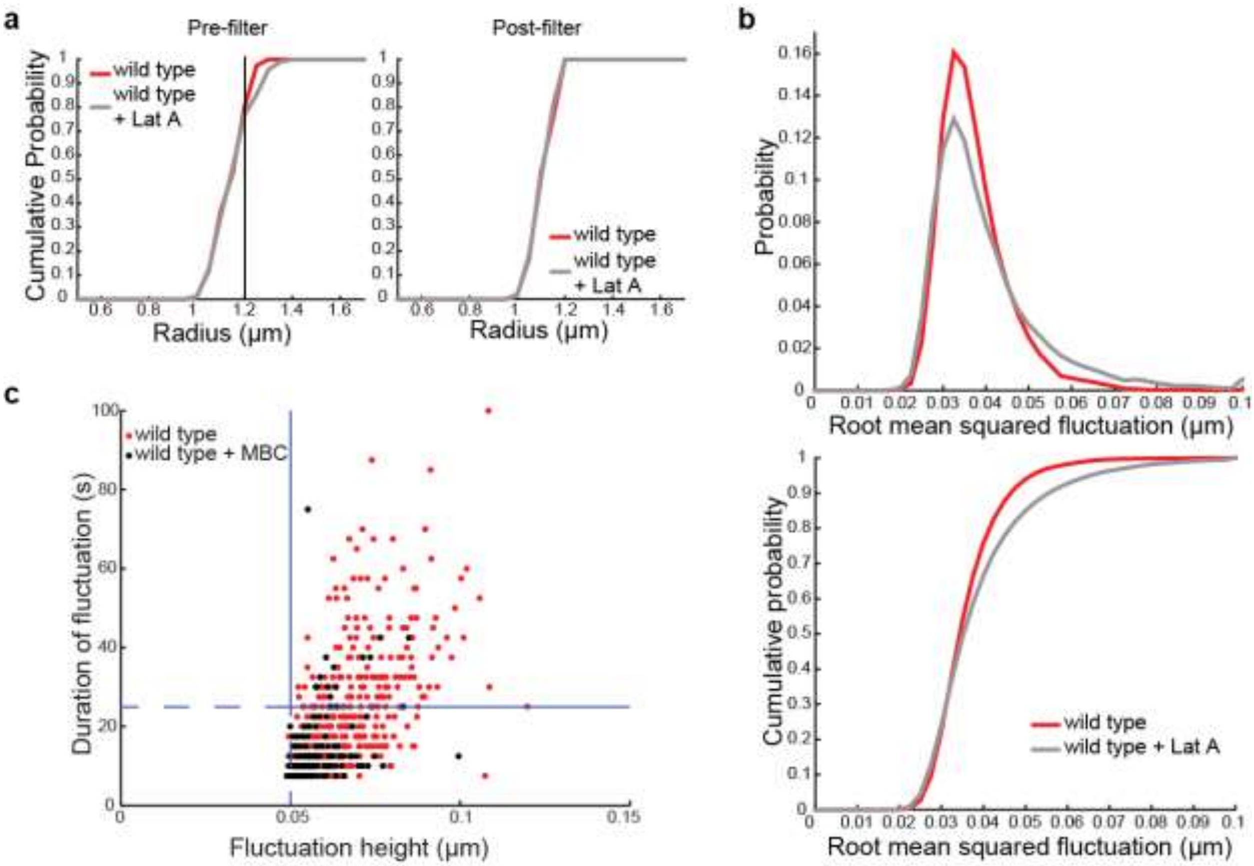

Supplementary Figure 1

(a) Cumulative probability plot of nuclear radius for wild type (red) and Lat A treated (grey) cells before (left) and after (right) size filter. Black line indicates the 1.2 μm threshold established for the population of Lat A treated cells arrested in G2. (b) Comparison of RMSF probability distribution (top) and cumulative probability distribution (bottom) between wild type (n=76) and Lat A treated (n=190) cells after imposing the size filter. (c) Plot of the duration of fluctuation times versus the fluctuation height for wild type cells either untreated (red) or treated with MBC (black). The blue lines indicate the cut-off established for a “large” fluctuation, which excludes greater than 90 percent of fluctuations seen in the presence of MBC. Data is from a minimum of three biological replicates.

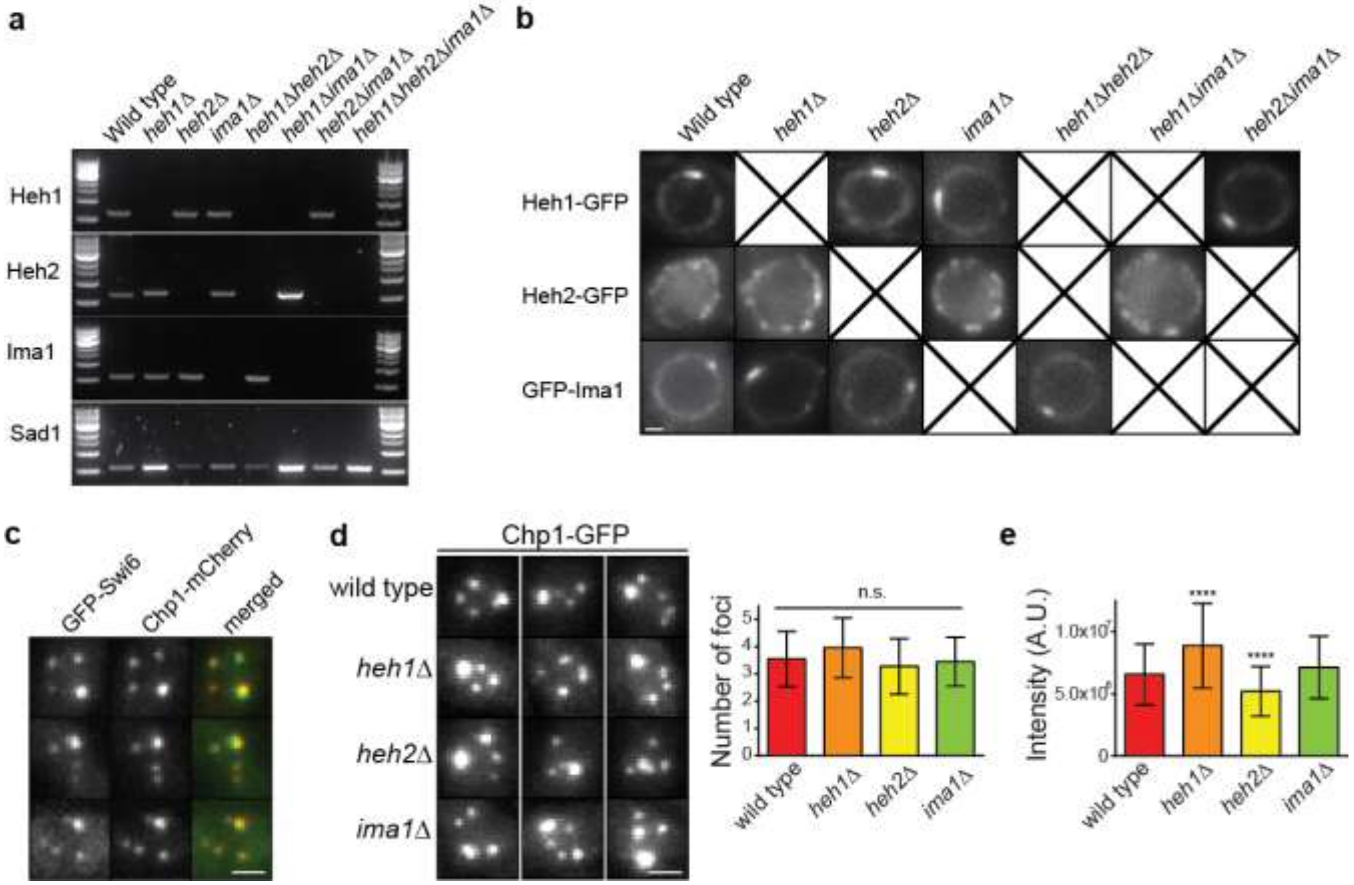

Supplementary Figure 2

(a) Validation of knock-out strain genotypes by PCR carried out on genomic DNA. Each product is internal to the gene indicated and is therefore absent in the knock-out strains. Primers used for PCR are listed in Supplementary Table 2. (b) Each INM protein localizes to the nuclear envelope independently. Localization of GFP fusions of Heh1, Heh2 or Ima1 in the absence of the other inner nuclear membrane proteins, as indicated. Scale bar equals 0.64 µm. (c) Fluorescence micrographs of wild type cells expressing Chp1-mCherry and GFP-Swi6 showing their co-localization in heterochromatic foci. Scale bar equals 1.6 µm. (d) Untethering chromatin from the nuclear periphery does not disrupt chromatin state. Representative images of Chp1-GFP (left) and quantification of the number of foci per nucleus (right) in wild type (n=1248), *heh1Δ* (n=794), *heh2Δ* (n=1075), and *ima1Δ* (n=891) strains. Plotted as the mean +/- s.d. Scale bar equals 1.6 µm. (e) Mean integrated fluorescence intensity of total focal Chp1-GFP per nucleus plotted +/- s.d. n.s. is not significant. **** p<0.0001 by student's t-test. Data from panels d and e are from one biological experiment.

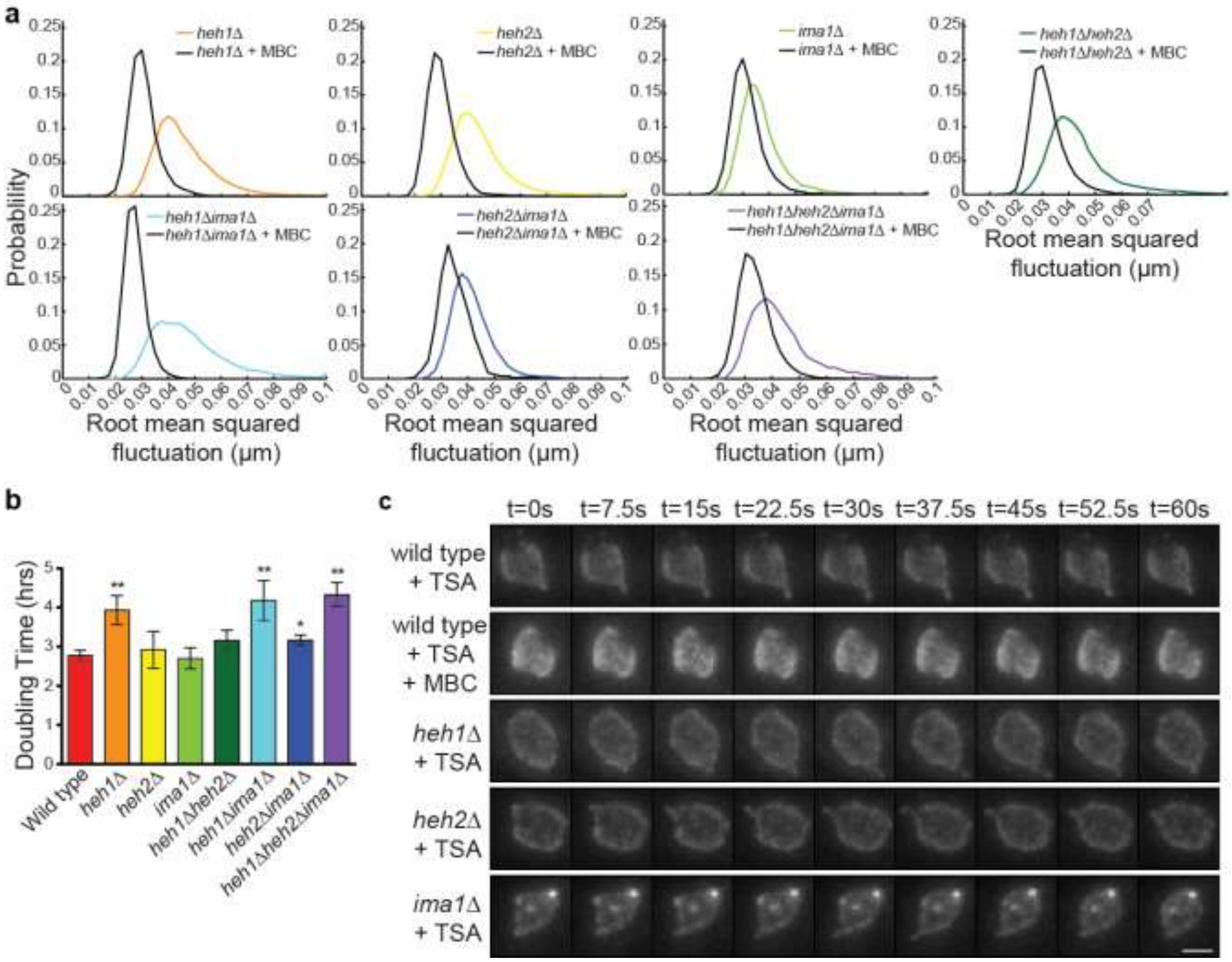

Supplementary Figure 3

(a) Comparison of the RMSF probability distribution for strains lacking each INM protein (or combinations of INM proteins) before (in colors) and after (in black) treatment with MBC. (b) Plot of the doubling time for each INM protein knock-out strain plotted as the mean +/- s.d. * $p<0.05$, ** $p<0.01$ by student's t-test. (c) Large, microtubule dependent nuclear envelope membrane tubules form in cells treated with TSA. Representative time courses from cells expressing Cut11-GFP and treated with TSA or TSA plus MBC. Images are maximum intensity projections from 10 z-slices with 400 nm spacing. Scale bar equals 1.6 μm. Data is from a minimum of three biological replicates.

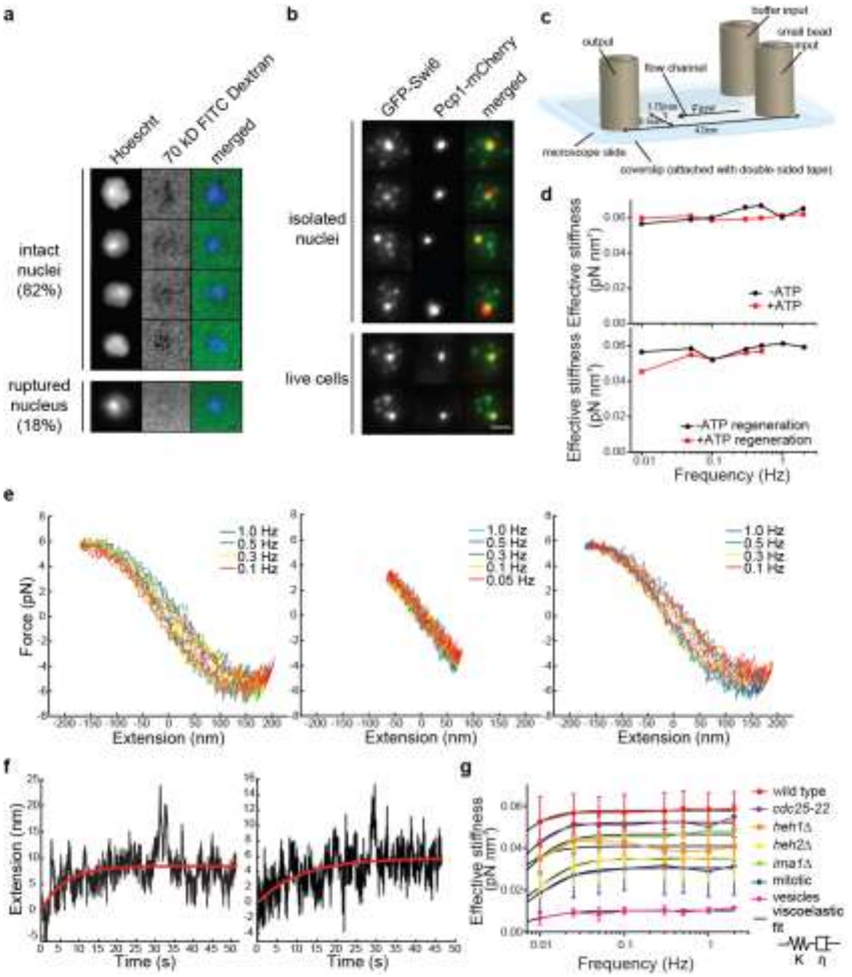

Supplementary Figure 4

(a) Representative images of intact wild type isolated nuclei (top) or ruptured isolated nuclei (bottom) in the presence of 70 kD FITC-dextran. DNA was labeled with Hoechst 33342. n = 150. Scale bar equals 1.6 µm. (b) Isolated nuclei have the same heterochromatin organization as intact cells. Representative images of isolated nuclei (top) and live cells (bottom) expressing GFP-Swi6, labelling the heterochromatin, and Pcp1-mCherry, labelling the spindle pole body. Scale bar equals 1.6 µm. (c) Diagram of flow cell used in optical tweezers experiments. (d) Nuclear stiffness of a wild type nucleus over a range of oscillation series, where the first round of oscillations was performed in the absence of ATP, and the second round of oscillations was performed in either 2 mM ATP (top) or 2 mM ATP + ATP regeneration system (bottom). (e) Force versus extension relationships from a single wild type nucleus. One set of 170 nm amplitude oscillation frequencies (left) were performed, followed by a set of 60 nm amplitude oscillation frequencies (middle) and then an additional round of 170 nm amplitude oscillation frequencies (right). (f) Additional examples of the creep response for two wild type nuclei acquired via a force clamp. The best fit parameters for the traces shown are $\Delta x = 9.2 \pm 0.3$ nm and $\tau = 11 \pm 1.4$ s (right) and $\Delta x = 8.0 \pm 0.4$ nm and $\tau = 6.2 \pm 1.1$ s (left). Errors represent the square root of the inverse observed Fisher Information. (g) Viscoelastic fits of optical tweezers data used to generate the data in Figure 5a-d. The effective stiffness of the nucleus was modeled with a Maxwell viscoelastic model, which consists of a spring and dashpot in series to account for the elastic component, *K*, and the viscous component, η, respectively. A non-linear least squares fit according to a Maxwell viscoelastic model, described in the Methods section, was applied to each time-dependent stiffness curve to extract the underlying elasticity and viscosity, which describe the plateau and divergence at lower frequencies, respectively.

Supplementary Table 1 – List of strains used in this paper

Strain (MKSP)	Genotype	Source
10	*cut11-GFP::ura4+ h+ ura4-D18 leu1-32*	Nurse Lab Collection;PN3779
438	*cdc25-22 cut11-GFP::ura4+ h- ura4-D18 leu1?*	this work
845	*heh2::natMX6 cut11-GFP::ura4+ h- ura4-D18 leu1-32*	this work
871	*ima1::natMX6 cut11-GFP::ura4+ h- ura4-D18 leu1-32*	this work
1148	*heh2::hphMX6 ima1::natMX6 cut11-GFP::ura4+ h- ura4-D18 leu1-32*	this work
1150	*heh1::kanMX6 cut11-GFP::ura4+ h-*	this work
1151	*heh1::kanMX6 heh2::hphMX6 cut11-GFP::ura4+ h+ ura4? leu1-32*	this work
1152	*heh1::kanMX6 ima1::natMX6 cut11-GFP::ura4+ h? ura4?*	this work
1153	*heh1::kanMX6 heh2::hphMX6 ima1::natMX6 cut11-GFP::ura4+ h+ ura4?*	this work
1172	*heh2::hphMX6 ima1::natMX6 heh1-GFP::kanMX6 h? ura4-D18 leu1-32*	this work
1173	*natMX6::nmt41GFP-ima1 sad1-mCherry::kanMX6 h? ura4?*	this work
1174	*heh1::kanMX6 heh2-GFP::hphMX6 h?*	this work
1399	*chp1-GFP::hphMX6 h+ ura4-D18 leu1-32*	this work
1415	*heh2::hphMX6 natMX6::nmt41GFP-ima1 h+ ura4? leu1?*	this work
1416	*ima1::natMX6 heh1-GFP::kanMX6 h? ura4-D19 leu1-32*	this work
1421	*chp1-mCherry::kanMX6 kanMX6::nmt41GFP-swi6 h? ura4-D18 leu1-32*	this work
1531	*heh1::kanMX6 natMX6::nmt41GFP-ima1 h?*	this work
1532	*ima1::natMX6 heh2-GFP::hphMX6 h? ura4?*	this work
1533	*heh1::kanMX6 ima1::natMX6 heh2-GFP::hphMX6 h+ ura4?*	this work
1735	*heh2::hphMX6 heh1-GFP::kanMX6 h? ura4? leu1?*	this work
1775	*heh2-GFP::hphMX6 sad1-mCherry::kanMX6 h? ura4? leu?*	this work
1776	*heh1-GFP::kanMX6 sad1-mCherry::hphMX6 h? ura4-D18 leu1-32*	this work
1804	*kanMX6::nmt41GFP-swi6 pcp1-mCherry::kanMX6 h+ ura4-D18 leu1-32*	this work
1898	*heh1::kanMX6 heh2::hphMX6 natMX6::nmt41GFP-ima1 h? ura4? leu1?*	this work
2015	*chp1-GFP::hphMX6 ima1::kanMX6 h? ura4-D18 leu1-32*	this work
2016	*chp1-GFP::hphMX6 heh2::natMX6 h? ura4-D18 leu1-32*	this work
2017	*chp1-GFP::hphMX6 heh1::kanMX6 h? ura4+ leu1+*	this work

Supplementary Table 2 – List of primers used for gene knock-out validation

**Primer name**	**Primer sequence**
Heh1intFchk	5' - GACAATTGGGAGGACCCTAACTTCGAATTGC - 3'
Heh1intRchk	5' - GAGGAGCAAACTCGATATCTCGAGAGACTC - 3'
Heh2intFchk	5' - GTCAACGGCGAAGAAAGCGCAGTTAATCAC - 3'
Heh2intRchk	5' - CGCCTAAGAAACTGAGAAAAGAACTTTGGTTC - 3'
Ima1intFchk	5' - GAGACGGAACGTCTACTTGGACTTGCTC - 3'
Ima1intRchk	5' - GTACCAATACGGGTTCCAAAAGACAACG -3'
Sad1AcidDomainFor	5' - GTTAGGAGCGAACTCTGCTCAGATTC - 3'
Sad1AcidDomainRev	5' - GGAATGTTTGGTACGGGCGAATTAGATG - 3'
